# New prospects for PET in prostate cancer imaging: a physicist's viewpoint

**DOI:** 10.1186/2197-7364-1-11

**Published:** 2014-09-09

**Authors:** Maurizio Conti

**Affiliations:** Siemens Healthcare Molecular Imaging, 810 Innovation Drive, Knoxville, TN 37932 USA

**Keywords:** Prostate cancer, PET, Time-of-flight, Detectability, Sensitivity, Spatial resolution

## Abstract

Prostate cancer is one of the most common forms of cancer among men. Early diagnosis, correct staging, accurate detection of metastasis, and monitoring of the therapy are the key tasks that could greatly benefit from medical imaging. After a review of the main developments in the field of positron emission tomography (PET) tracers for prostate cancer, the impact of improved PET instrumentation with good spatial resolution and high sensitivity is discussed, together with the latest development in PET technology: lutetium oxy-ortho-silicate (LSO) and lutetium-yttrium oxy-ortho-silicate (LYSO) scintillators, resolution recovery, and time-of-flight reconstruction. New directions and multiple approaches in PET instrumentation for prostate cancer are presented and discussed. In particular, improved hardware and noise suppressing reconstruction algorithms allow for higher detectability of small lesions and better spatial resolution in PET/computerized tomography (CT) and PET/magnetic resonance (MR). This can be beneficial for guiding biopsy and surgery and for accurate therapy monitoring.

**Electronic supplementary material:**

The online version of this article (doi:10.1186/2197-7364-1-11) contains supplementary material, which is available to authorized users.

## Introduction

Medical imaging techniques are used in prostate cancer (PCa) for diagnosis, staging, detection of local recurrence and metastasis, and therapy monitoring. They include ultrasound (US), computerized tomography (CT), planar bone scintigraphy, single photon emission computed tomography (SPECT), positron emission tomography (PET), and magnetic resonance imaging (MRI). In the last decade, there has been an increase of the use of PET for prostate cancer and a greater interest in investigating PET imaging capabilities for this application [1–7]. The increased use of PET still requires the development of a new tracer that is both sensitive and specific for prostate cancer. Assuming such an agent will be developed and there are several candidates, imaging of prostate cancer will be challenging and will probably require additional improvements in instrumentation.

The first step in the diagnosis of prostate cancer is often an anomalous prostate-specific antigen (PSA) value. The PSA test is not specific for cancer, lacks in the ability to differentiate low-grade and high-grade cancer, and is not able to localize the tumor. Typically, after an elevated serum PSA, a blind biopsy guided by transrectal ultrasound is performed. The biopsy is ‘blind’ as it systematically places needles within the prostate rather than guiding the needles to specific abnormalities. It has been observed that this biopsy may detect only 50% of the clinically significant malignant areas found on histological examination of the whole prostate (after removal of the prostate) [8, 9]. The ability to reliably assess the location and the aggressiveness of prostate cancer without removing the prostate could have a strong impact on the choice of patient treatment.

Once the prostate cancer has been diagnosed, the typical treatment options for organ-confined prostate cancer are radical prostatectomy, radiation therapy, and active surveillance. More precise localization of the tumors would improve treatment for all these options. For instance, surgeons could spare critical nerves in areas of low risk and perform wider resections near the tumor. For radiation therapy (RT), correct localization of the tumor could improve the delivery of radiation to increase both the safety and the efficacy of this treatment [10, 11]. However, both surgery and RT are not without significant risk of clinical side effects. Because urinary incontinence and erectile dysfunction are not uncommon after radical prostatectomy and RT, there is also a growing concern regarding the risk for overdiagnosis and, consequently, overtreatment of potentially indolent disease. Since most of the patients with non-aggressive disease might not experience clinical effects of prostate cancer in their lifetime, a technique called ‘active surveillance’ can be used: no treatment is adopted, but the cancer is monitored as closely as possible to be able to intervene as soon as needed. For patients choosing active surveillance, non-invasive localization and characterization of the tumor could allow for monitoring of the lesion and moving to an active treatment only if and when needed. Accurate imaging techniques could be very important to monitor a possible evolution of the disease under active surveillance.

Despite best efforts, about 10% of men progress to metastatic disease. Typically, prostate cancer metastases occur first in the pelvic lymph nodes and bone. Early lymph node involvement is especially hard to detect by conventional imaging methods such as ultrasound, CT, or MRI, because the metastasis in lymph nodes is small and grows slowly, making it difficult to diagnose positive nodes on the basis of enlargement of the nodes. Therefore, pelvic lymph node dissection is often necessary, with associated risks [1]. For bone metastasis, bone scintigraphy is used, but it is non-specific and characterized by poor spatial resolution.

## Review

### PET and PET tracers for prostate cancer imaging

PET (and PET/CT) has been used as a method for prostate tumor localization. The standard oncology PET tracer, ^18^F-FDG, has been disappointing for early detection and localization of primary PCa because of high bladder activity, relatively low tumor uptake, and low specificity [1, 6, 7, 12]. Alternative PET tracers that exhibit higher sensitivity and slightly higher specificity have been used, such as ^11^C-labeled and ^18^F-labeled choline and acetate [13]. The lack of specificity of the most common PET tracers and the poor resolution of the PET cameras are seen as the major limitations of PET imaging [3, 5, 14]. Another key limitation, common to PET and all other imaging techniques, is the inability to discriminate between indolent and aggressive disease [5]. In Table 1, a list of the main PET tracers, available or under development and test, is shown, and a brief review of their characteristics is presented in this section.

**Table 1:** **PET tracers for prostate cancer**

Tracer	Mechanism	Specificity
^18^F-FDG	Glucose metabolism	Non-specific
^11^C/^18^F-choline	Lipid metabolism	Non-specific
^11^C/^18^F-acetate	Lipid metabolism	Non-specific
^18^F-NaF	Calcium analog	Non-specific
^11^C-methionine	Amino acid transport	Non-specific
^18^F-FACBC	Amino acid transport	Non-specific
^18^F-FLT	Cell proliferation	Non-specific
^18^F-FMAU	Cell proliferation	Non-specific
^18^F-FDHT	Androgen receptor	Specific
^18^F-DCFBC, ^64^Cu/^89^Zr-J591, ^68^Ga-PSMA, others	PSMA inhibitors/antibodies	Specific

#### ***Imaging with***^***18***^***F-FDG***

^18^F-FDG has low uptake and low sensitivity in the primary stage of PCa and in the pelvic lymph nodes; moreover, it does not differentiate benign prostate hyperplasia, postoperative scarring, and malignant tumors [12, 15]. On the other hand, it has shown some capability in imaging and assessment of treatment response in advanced castration-resistant (both to medical and surgical treatments) prostate cancer [6, 12].

#### Imaging of phospholipids (choline)

Choline is a component of biologic membranes. Malignant tumors show increased demand for cell membrane synthesis and, accordingly, an increased uptake of choline [6, 13]. The main PET tracers based on choline are ^11^C-choline [16, 17], ^18^F-fluoroethylcholine (FEC) [18], and ^18^F-fluorocholine (FCH) [19]. Urinary excretion of ^18^F-choline is higher than that of ^11^C-choline, but overall imaging methods and results are similar between different choline agents. The main difference is the half-life: the ^11^C-choline half-life is 20 min, and the ^18^F-choline half-life is 110 min. This agent has been in development for at least 10 years and is engaged in research facilities around the world.

#### Imaging of fatty acid synthesis (acetate)

Prostate cancer itself is associated with an increase in fatty acid synthesis. A high concentration of ^11^C-acetate has been seen in prostate cancer. This tracer also has the benefit of not being excreted by the kidneys, making it preferable to ^18^F-FDG for visualizing pelvic disease but suffers from the short half-life of ^11^C [20, 21]. ^18^F-acetate is also available but has not been widely applied.

#### ***Imaging of bone metastasis activity with***^***18***^***F-fluoride***

^18^F-fluoride (typically NaF) is a high-sensitivity tracer for the detection of bone metastases in patients with prostate cancer, but it is not tumor specific [22].

#### Amino acid transport imaging and cell proliferation

Uptake of ^11^C-labeled methionine is associated with amino acid transport and protein synthesis during tumor proliferation. Methionine is rapidly cleared from the blood and is metabolized in the liver and pancreas without renal excretion, making it more suitable than ^18^F-FDG for imaging pelvic disease [4, 7]. Another tracer that follows the amino acid transport mechanism is ^18^F-FACBC which is a radiolabeled analog of leucine [23]. Other tracers associated with cell proliferation have been used, such as ^18^F-FLT and ^18^F-FMAU [6]. All these tracers are not prostate cancer specific.

#### Imaging of androgen receptor expression

^18^F-fluoro-5a-dihydrotestosterone (FDHT) is a radiolabeled analog of dihydrotestosterone, the main androgen receptor ligand. The androgen receptor plays a major role in PCa growth. FDHT is typically used to monitor metastasis in advanced disease and has specifically been used to develop new hormone therapies for treating cancer [6, 7].

#### New PCa-specific tracers

A new generation of PCa-specific tracers is being developed and tested that target tumor antigens that are unique to prostate cancer. These include ligands such as antibodies and small molecules that bind to specific sites associated with PCa growth; for example, the prostate-specific membrane antigen (PSMA). Most new tracers are presently in the animal testing phase or early human testing phase. PSMA and PSA are being targeted with antibodies, such as ^89^Zr-J591 or ^64^Cu-J591 for PSMA [24] and ^89^Zr-5A10 for PSA [25]. Other PSMA ligands are ^68^Ga-PSMA [26] and ^18^F-DCFBC [27]; both tracers are already being tested on patients. Animal studies showed excellent imaging capabilities of ^124^I and ^18^F minibodies binding to the prostate stem cell antigen (PSCA) [28, 29].

### Need and value of new PET technologies

In addition to the lack of a widely available PET imaging probe for prostate cancer, another problem is that prostate cancer, at its early stages, tends to be small within the prostate, within lymph nodes, and within early bone metastases. A high-resolution, high-sensitivity, and high-specificity method to accurately localize cancer within the prostate and in the pelvic region would be highly beneficial. Since new more specific PET tracers are being developed and tested, the need for better instrumentation becomes stronger, in particular, the need for PET cameras with few millimeter resolution [5, 11, 14]. Apart from the lack of a specific tracer, the noise level and the spatial resolution have been limiting the proficient use of PET in prostate cancer diagnosis and treatment monitoring. A review of the scientific literature shows that published studies with fluorodeoxyglucose (FDG), choline, and acetate were limited to lesions larger than 5 mm [5, 12, 14], and often poor performance was observed for lesions smaller than 9 mm [30] or even less than 2 cm in the presence of high noise or background [14].

**Figure 1:**
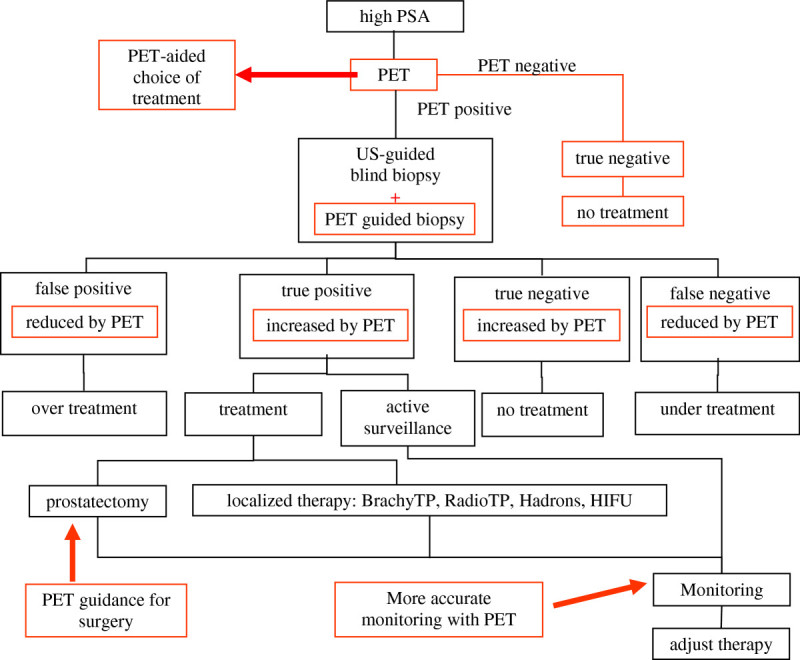
**A flow diagram of prostate cancer diagnosis and treatment.** Also shown is the change of action and outcome if ‘improved PET’ imaging techniques (red) are used.

High-sensitivity, high-resolution molecular imaging instrumentation, coupled with the new high-uptake and high-specificity molecular agents, can provide help for:

-  Guiding the biopsy and reducing understaging (and overstaging) and treatment;
-  Monitoring the untreated tumor under ‘active surveillance’;
-  Guiding prostatectomy, reducing positive margins, and sparing healthy tissue;
-  Reducing the need of surgical removal of pelvic lymph nodes;
-  Monitoring response to therapy; and
-  Detecting metastasis at an early stage.In Figure 1, a flow diagram of prostate cancer diagnosis and treatment is shown, with the possible positive effect of an improved PET imaging technique (red).

The increasing availability of a new generation of PET scanners in the clinical environment makes it now possible to revisit the limitations of PET. The new PET scanners have higher sensitivity and improved reconstruction algorithms: both factors contribute to lowering of the noise level and allow for the exploitation of the full spatial resolution of the PET scanner. The impact of new PET technologies has not been fully assessed in this field.

The following innovations can have a positive impact on prostate cancer imaging:

-  new scintillation materials: lutetium oxy-ortho-silicate (LSO) and lutetium-yttrium oxy-ortho-silicate (LYSO) → higher sensitivity
-  smaller detector crystals → higher resolution
-  longer axial coverage (>20 cm) → higher sensitivity
-  resolution recovery reconstruction → lower noise, higher contrast
-  time-of-flight reconstruction → lower noise, higher sensitivity
-  new development of PET/MR → multimodality synergy

From the point of view of PET image reconstruction, two innovations can be underlined as driving the present evolution: resolution recovery (or point spread function) reconstruction and time-of-flight reconstruction. Point spread function reconstruction (PSF) is characterized by significant noise reduction and contrast enhancement [31]. Time-of-flight reconstruction (TOF) allows for faster convergence and reduced noise propagation; it is less sensitive to imprecise attenuation and scatter correction, and it works as a virtual sensitivity amplifier [32, 33]. The combination of these techniques has had a significant impact on image quality and detectability of cancer lesions [34–37]. In particular, the noise reduction and virtual count amplification offered by the PSF + TOF reconstruction and the actual increase of sensitivity offered by LSO and larger field of view (FOV) could allow for a smaller pixel size in the reconstructed image and eliminate or reduce the need for image smoothing.

**Figure 2:**
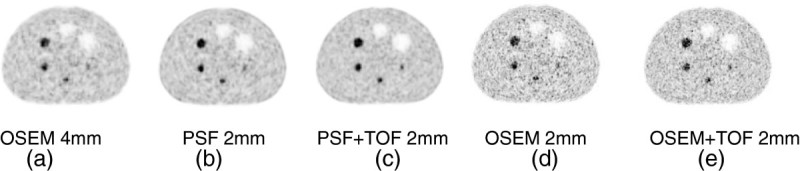
**Transaxial slice of an image quality phantom**, **scanned on a Siemens mCT, reconstructed using different methods. (a)** A standard iterative algorithm (OSEM) at typical pixel size of 4 mm, **(b)** OSEM + PSF with 2-mm pixel size, **(c)** OSEM + PSF + TOF with 2-mm pixel size, **(d)** OSEM with 2-mm pixel size, and **(e)** OSEM + TOF with 2-mm pixel size.

An example of a possible new direction for high-resolution imaging is provided in Figure 2. An image quality phantom, scanned on a Siemens mCT PET scanner (Siemens AG, Munich, Germany) [38], has been reconstructed using a standard iterative algorithm, ordered subset expectation maximization (OSEM), with typical image pixel size of 4 mm (a) and using OSEM with PSF and TOF, with smaller pixel size of 2 mm. The scan contains 26 × 10^6^ total true counts, equivalent to a typical FDG oncology study. Conventional OSEM with a smaller pixel size results in high noise and poor image quality (d). Adding only PSF allows for imaging with a 2-mm pixel size, with acceptable noise level and slightly improved resolution (b). Adding only TOF produces a higher resolution image with higher noise level (e). But the combined effect of PSF and TOF allows for lower background noise and improved spatial resolution (c). In fact, this detectability improvement with smaller pixel size and advanced reconstruction has been observed by other groups [39].

Given this new landscape of PET instrumentation development, at least three approaches could be taken in order to improve spatial resolution and sensitivity of PET scanners for prostate cancer imaging: (1) a standard whole-body PET scanner, with PSF and TOF reconstruction, and a reconstruction protocol optimized for prostate cancer; (2) a standard large-ring PET scanner with a high-resolution insert [40–43], with a local magnification effect [44, 45]; and (3) a dedicated small-diameter PET camera with small scintillating crystals, possibly in multimodality [46–48].

Each of these approaches has advantages and disadvantages. The first option, using the new generation of TOF PET scanners at full resolution, without any insert, does not require any development effort, but probably cannot achieve the same results as the other two solutions, in terms of spatial resolution. The PET insert in a standard PET scanner could take advantage of the large installed base already available in hospitals and improve the local spatial resolution, but a considerable effort should be put into reconstruction development for the new geometry. A dedicated camera can be more compact and easy to use and has a lower cost but would require a large demand and engineering effort to become a reliable standard.

It is already possible to assess the improvement opportunities associated with the first approach, using simulation and reprocessing experimental data from clinical sites, acquired in the past with non-TOF PET scanners. In an ongoing study at Siemens Healthcare Molecular Imaging, a set of clinical PET images of patients with prostate cancer, acquired using ^11^C-choline and ^11^C-acetate, were selected as a starting point for a simulation [49]. Small lesions were added via software in selected locations, with variable size and intensity. Then, the 3D images were forward projected into a sinogram space, assuming a TOF capability, using the sinogram representation of a Siemens mCT TOF PET scanner [38]. In the process, detector sensitivity, attenuation, scatter, randoms, and Poisson noise were added. The data were reconstructed with different reconstruction methods.

**Figure 3:**
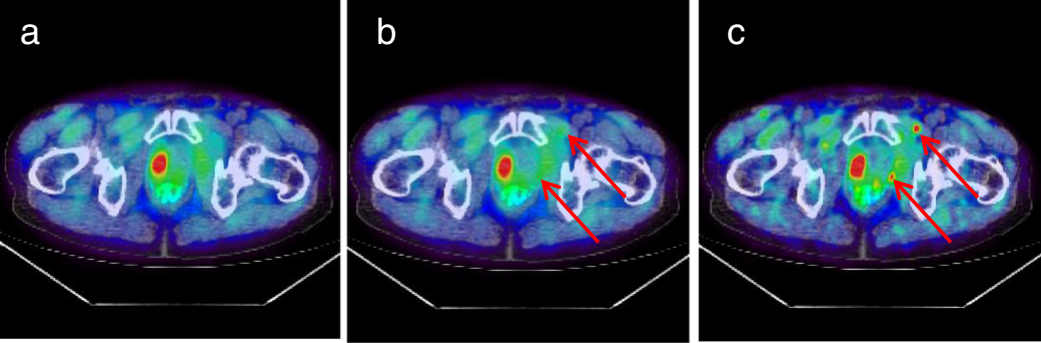
**A prostate cancer patient with**^**11**^ **C-choline injection. (a)** The original fused PET/CT image, with no simulated lesion (the ‘true’ original large tumor is visible in the prostate); **(b)** the OSEM reconstruction with the same parameters as in the original image (5.5 × 5.5 × 3.3 mm^3^ voxels, OSEM with 20 subsets, 2 iterations, and a 6-mm post-reconstruction filter), after insertion of two 6 mm lesions with SUV = 8; **(c)** the PSF + TOF reconstruction with 2-mm voxel size and 4-mm filter, after insertion of the two 6-mm lesions. The arrows point to the location of the simulated lesions.

A fused PET/CT transaxial slice of a sample patient, scanned on a BGO-based PET/CT with ^11^C-choline as a tracer, is presented in Figure 3. The patient, weight 65 kg, was injected with 295 MBq of ^11^C-choline, and acquisition time was 120 s. The patient, imaged before a prostatectomy, was diagnosed with prostate cancer that was localized in the prostate. The original image had 5.5 × 5.5 × 3.3 mm^3^ voxels, the reconstruction method was OSEM with 20 subsets, 2 iterations, and a 6-mm post-reconstruction filter (Figure 3a). Two simulated lesions were inserted in the pelvic region: the size of both lesions was 6 mm, and the simulated SUV was 8. One can appreciate the improved visual detectability using PSF + TOF and 2-mm pixel size and 4-mm filter (Figure 3c) over the original reconstruction method (Figure 3b). The two lesions, not visible at low resolution, are clearly visible using PSF + TOF and high resolution.

These images, based on simulations of realistic distributions of prostate cancer PET tracers, hint that it is possible to push the past limits of detectability and localization of small tumors in the pelvic area, if using the present generation of TOF PET scanners. This needs to be confirmed by experimental data. Some clinical studies are already showing a marked improvement in detecting small metastatic lesions in prostate cancer [50]. Also, PET images have to be correlated with post-surgery histological examinations to verify the PET capability to correctly identify the spatial distribution of cancerous cells and its concentration. A recent simulation study attempted to quantify the smallest detectable activity in a prostate cancer small lesion or metastasis [49]. Such smallest detectable activity can be correlated with number of cancer cells in the lesion. If experimental studies could confirm minimum detectable activity, and if such limit were acceptable compared to the accuracy of the histological examination, then one could think of replacing lymph node surgery with non-invasive imaging. Up to now, clinical studies aimed to assess the value of PET/CT for preoperative nodal staging were disappointing or only partially encouraging [51, 52]; on the other hand, the new generation of TOF scanners were not used in those studies. In general, it needs to be assessed whether we can reduce false negatives and false positives using better reconstruction and what kind of support the improved spatial localization and improved small-tumor detectability can provide to biopsy and/or surgery.

The other key issue in prostate cancer diagnosis and therapy, together with detectability and accurate localization, is the tumor characterization and the non-invasive discrimination of aggressive from indolent disease. In this field, the new PET/MR multimodality could be very instrumental [53–55]. As PET/CT has shown in the past decade, two combined modalities are a powerful tool to improve accuracy and specificity in the diagnosis, and MR has superior capability in imaging soft tissue, as compared to CT. There is evidence that PET and MR imaging, and multiparametric MR in particular [56–58], reinforce each other and improve the reliability of the diagnosis [30, 59, 60]. From the point of view of PET, few studies are available that attempt to correlate the kinetic parameters of the PET tracer or the SUV values with aggressiveness of the disease [21, 23, 61]. Past results are sometimes contradictory for choline and acetate, and the newer tracers still need to be fully characterized. Higher sensitivity PET scanners and improved reconstruction algorithms offer lower noise and better accuracy, and this could be beneficial also for tracer kinetics studies, possibly reducing measurement uncertainty and providing clearer results. Multiparametric MRI and hybrid techniques combining PET and MRI parameters have shown some improvement in discriminating aggressive from non-aggressive disease [62], and, at least, multiparametric MRI can provide information about the risk of aggressiveness [58]. In particular, magnetic resonance spectroscopy imaging (MRSI) has been used for tumor characterization: the relative ratio of (choline + creatine) over citrate has been seen as a marker of aggressiveness [59, 63]. A recent study on a PET/MR scanner using ^18^F-choline showed that multiparametric MRI, coupled with PET, enhances sensitivity and specificity of the single modalities, and PET SUV values were found to be better correlated with high Gleason score than the results of the blind biopsy [60]. In conclusion, improved technologies and multimodalities, such as multiparametric MR + PET and dynamic PET, could be useful investigative tools, together with more specific PET tracers, in the search for markers of aggressiveness [64].

## Conclusions

Prostate cancer diagnosis and treatment can greatly benefit from improved imaging techniques and from PET in particular. The present research emphasis on new specific tracers and the increasing availability of a new generation of PET scanners in the clinical environment makes it now possible to revisit the limitations of PET. The new PET scanners have higher sensitivity and improved reconstruction algorithms: both factors contribute to lowering of the noise level and allow for the exploitation of the full spatial resolution of the PET scanner. Higher detectability of small lesions and better spatial resolution in PET/CT and PET/MR can be beneficial for guiding biopsy and surgery and for accurate therapy monitoring. PET/MR, with the support of multiparametric MRI, could be instrumental to investigate aggressiveness of the disease.

Today, PET and medical physicists, by optimizing present PET scanner protocols, exploring new technologies and new multimodalities, and working on new and dedicated architectures, have a unique opportunity to support physicians and radiotracer scientists in the quest for a better diagnosis and treatment of prostate cancer.

## Authors’ original submitted files for images

Below are the links to the authors’ original submitted files for images. Authors’ original file for figure 1
Authors’ original file for figure 2
Authors’ original file for figure 3
Authors’ original file for figure 4
Authors’ original file for figure 5
Authors’ original file for figure 6

## References

[1] Kotzerke J, Gschwend JE, Neumaier B (2002). PET for prostate cancer imaging: still a quandary or the ultimate solution?. J Nucl Med.

[2] Zaheer A, Cho SY, Pomper MG (2009). New agents and techniques for imaging prostate cancer. J Nucl Med.

[3] Farsad M, Schiavina R, Franceschelli A, Sanguedolce F, Castellucci P, Bertaccini A, Brunocilla E, Manferrari F, Concetti S, Garofalo M, Rocca C, Borghesi M, Franchi R, Fanti S, Nanni C, Martorana G (2008). Positron-emission tomography in imaging and staging prostate cancer. Cancer Biomark.

[4] Apolo AB, Pandit-Taskar N, Morris MJ (2008). Novel tracers and their development for the imaging of metastatic prostate cancer. J Nucl Med.

[5] Fox JJ, Schoder H, Larson SM (2012). Molecular imaging of prostate cancer. Curr Opin Urol.

[6] Jadvar H (2012). Molecular imaging of prostate cancer: PET radiotracers. AJR Am J Roentgenol.

[7] Lutje S, Boerman OC, Van Rij CM, Sedelaar M, Helfrich W, Oyen WJ, Mulders PF (2012). Prospects in radionuclide imaging of prostate cancer. Prostate.

[8] Kwee SA, Wei H, Sesterhenn I, Yun D, Coel MN (2006). Localization of primary prostate cancer with dual-phase ^18^F-fluorocholine PET. J Nucl Med.

[9] Wefer AE, Hricak H, Vigneron DB, Coakley FV, Lu Y, Wefer J, Mueller-Lisse U, Carroll PR, Kurhanewicz J (2000). Sextant localization of prostate cancer: comparison of sextant biopsy, magnetic resonance imaging and magnetic resonance spectroscopic imaging with step section histology. J Urol.

[10] Picchio M, Giovannini E, Crivellaro C, Gianolli L, Di Muzio N, Messa C (2010). Clinical evidence on PET/CT for radiation therapy planning in prostate cancer. Radiother Oncol.

[11] Bouchelouche K, Tagawa ST, Goldsmith SJ, Turkbey B, Capala J, Choyke P (2011). PET/CT imaging and radioimmunotherapy of prostate cancer. Semin Nucl Med.

[12] Jadvar H (2011). Prostate cancer: PET with ^18^F-FDG, ^18^F- or ^11^C-acetate, and ^18^F- or ^11^C-choline. J Nucl Med.

[13] Schoeder H, Larson SM (2004). Positron emission tomography for prostate cancer. Semin Nucl Med.

[14] De Jong IJ, Pruim J, Elsinga PH, Vaalburg W, Mensink HJ (2003). Preoperative staging of pelvic lymph nodes in prostate cancer by ^11^C-choline PET. J Nucl Med.

[15] Dimitrakopoulou-Strauss A, Strauss LG (2003). PET imaging of prostate cancer with ^11^C-acetate. J Nucl Med.

[16] Picchio M, Treiber U, Beer AJ, Metz S, Bossner P, Van Randenborgh H, Paul R, Weirich G, Souvatzoglou M, Hartung R, Schwaiger M, Piert M (2006). Value of ^11^C-choline PET and contrast-enhanced CT for staging of bladder cancer: correlation with histopathologic findings.. J Nucl Med.

[17] Reske SN, Blumstein NM, Neumaier B, Gottfried HW, Finsterbusch F, Kocot D, Moeller P, Glatting G, Perner S (2006). Imaging prostate cancer with ^11^C-choline PET/CT.. J Nucl Med.

[18] Hara T, Kosaka N, Kishi H (2002). Development of ^18^F-fluoroethylcholine for cancer imaging with PET: synthesis, biochemistry, and prostate cancer imaging. J Nucl Med.

[19] Degrado TR, Coleman RE, Wang S, Baldwin SW, Orr MD, Robertson CN, Polascik TJ, Price DT (2000). Synthesis and evaluation of ^18^F-labeled choline as an oncologic tracer for positron emission tomography: initial findings in prostate cancer.. Cancer Res.

[20] Oyama N, Miller TR, Dehdashti F, Siegel BA, Fischer KC, Michalski JM, Kibel AS, Andriole GL, Picus J, Welch MJ (2003). ^11^C-acetate PET imaging of prostate cancer: detection of recurrent disease at PSA relapse. J Nucl Med.

[21] Schiepers C, Hoh CK, Nuyts J, Seltzer M, Wu C, Huang SC, Dahlbom M (2008). ^11^C-acetate kinetics of prostate cancer. J Nucl Med.

[22] Even-Sapir E, Metser U, Mishani E, Lievshitz G, Lerman H, Leibovitch I (2006). The detection of bone metastases in patients with high-risk prostate cancer: ^99m^Tc-MDP planar bone scintigraphy, single- and multi-field-of-view SPECT, ^18^F-fluoride PET, and ^18^F-fluoride PET/CT. J Nucl Med.

[23] Schuster DM, Votaw JR, Nieh PT, Yu W, Nye JA, Master V, Bowman FD, Issa MM, Goodman MM (2007). Initial experience with the radiotracer anti-1-amino-3-18F-fluorocyclobutane-1-carboxylic acid with PET/CT in prostate carcinoma. J Nucl Med.

[24] Holland JP, Divilov V, Bander NH, Smith-Jones PM, Larson SM, Lewis JS (2010). ^89^Zr-DFO-J591 for immunoPET of prostate-specific membrane antigen expression in vivo. J Nucl Med.

[25] Ulmert D, Evans MJ, Holland JP, Rice SL, Wongvipat J, Pettersson K, Abrahamsson PA, Scardino PT, Larson SM, Lilja H, Lewis JS, Sawyers CL (2012). Imaging androgen receptor signaling with a radiotracer targeting free prostate-specific antigen. Cancer Discov.

[26] Afshar-Oromieh A, Malcher A, Eder M, Eisenhut M, Linhart HG, Hadaschik BA, Holland-Letz T, Giesel FL, Kratochwil C, Haufe S, Haberkorn U, Zechmann CM (2013). PET imaging with a [^68^Ga]gallium-labelled PSMA ligand for the diagnosis of prostate cancer: biodistribution in humans and first evaluation of tumour lesions. Eur J Nucl Med Mol Imaging.

[27] Mease RC, Dusich CL, Foss CA, Ravert HT, Dannals RF, Seidel J, Prideaux A, Fox JJ, Sgouros G, Kozikowski AP, Pomper MG (2008). *N*-[*N*-[(*S*)-1,3-dicarboxypropyl]carbamoyl]-4-[^18^F]fluorobenzyl-l-cysteine, [^18^F]DCFBC: a new imaging probe for prostate cancer.. Clin Cancer Res.

[28] Wu AM (2009). Antibodies and antimatter: the resurgence of immuno-PET. J Nucl Med.

[29] Wu AM (2014). Engineered antibodies for molecular imaging of cancer. Methods.

[30] Mena E, Turkbey B, Mani H, Adler S, Valera VA, Bernardo M, Shah V, Pohida T, McKinney Y, Kwarteng G, Daar D, Lindenberg ML, Eclarinal P, Wade R, Linehan WM, Merino MJ, Pinto PA, Choyke PL, Kurdziel KA (2012). ^11^C-acetate PET/CT in localized prostate cancer: a study with MRI and histopathologic correlation. J Nucl Med.

[31] Panin VY, Kehren F, Michel C, Casey M (2006). Fully 3-D PET reconstruction with system matrix derived from point source measurements. IEEE Trans Med Imaging.

[32] Conti M (2009). State of the art and challenges of time-of-flight PET. Phys Med.

[33] Conti M (2011). Focus on time-of-flight PET: the benefits of improved time resolution. Eur J Nucl Med Mol Imaging.

[34] Kadrmas DJ, Casey ME, Conti M, Jakoby BW, Lois C, Townsend DW (2009). Impact of time-of-flight on PET tumor detection. J Nucl Med.

[35] Karp JS, Surti S, Daube-Witherspoon ME, Muehllehner G (2008). Benefit of time-of-flight in PET: experimental and clinical results. J Nucl Med.

[36] Lois C, Jakoby BW, Long MJ, Hubner KF, Barker DW, Casey ME, Conti M, Panin VY, Kadrmas DJ, Townsend DW (2010). An assessment of the impact of incorporating time-of-flight information into clinical PET/CT imaging. J Nucl Med.

[37] Schaefferkoetter J, Casey M, Townsend D, El Fakhri G (2013). Clinical impact of time-of-flight and point response modeling in PET reconstructions: a lesion detection study. Phys Med Biol.

[38] Jakoby B, Bercier Y, Conti M, Casey M, Bendriem B, Townsend D (2011). Physical and clinical performance of the mCT time-of-flight PET/CT scanner. Phys Med Biol.

[39] Morey AM, Noo F, Kadrmas DJ, Choi Y (2013). Improved PET lesion-detection performance using 2mm pixels. IEEE Nuclear Science Symposium and Medical Imaging Conference.

[40] Clinthorne N, Brzezinski K, Chesi E, Cochran E, Grkovski M, Grošičar B, Honscheid K, Huh S, Kagan H, Lacasta C, Linhart V, Mikuž M, Smith S, Stankova V, Studen A, Weilhammer P, žontar D (2013). Silicon as an unconventional detector in positron emission tomography. Nucl Instrum Methods Phys Res A.

[41] Garibaldi F, De Leo R, Ranieri A, Loddo F, Floresta M, Tamma C, Gabrielli A, Giorgi F, Cusanno F, Musico P, Perrino R, Finocchiaro P, Cosentino L, Pappalardo A, Meddi F, Maraviglia B, Giove F, Gili T, Capuani S, Turisini M, Clinthorne N, Huh S, Majewski S, Lucentini M, Gricia M, Giuliani F, Monno E, Ziock K (2010). TOPEM: a multimodality probe (PET TOF, MRI, and MRS) for diagnosis and follow up of prostate cancer. IEEE Nuclear Science Symposium Conference Record.

[42] Janecek M, Wu H, Tai Y-C, Seibert JA (2004). High resolution insert for clinical whole body PET scanners: design and optimization. IEEE Nuclear Science Symposium Conference Record.

[43] Stolin AV, Majewski S, Jaliparthi G, Raylman RR (2013). Construction and evaluation of a prototype high resolution, silicon photomultiplier-based, tandem positron emission tomography system. IEEE Trans Nucl Sci.

[44] Tai YC, Wu H, Pal D, O'Sullivan JA (2008). Virtual-pinhole PET. J Nucl Med.

[45] Zhou J, Qi J (2009). Theoretical analysis and simulation study of a high-resolution zoom-in PET system. Phys Med Biol.

[46] Huber JS, Moses WW, Pouliot J, Hsu I, Seibert JA (2004). Dual-modality PET/ultrasound imaging of the prostate. IEEE Nuclear Science Symposium Conference Record.

[47] Majewski S, Stolin A, Delfino E, Martone P, Proffitt J, Chmeissani M (2011). High resolution fast stereotactic PET imager for prostate biopsy. IEEE Nuclear Science Symposium and Medical Imaging Conference.

[48] Turkington TG, Hawk T, Coleman R, Smith M, Majewski S, Kross B, Wojcik R, Weisenberger AG, DeGrado TR, Coleman RE, Seibert JA (2004). PET prostate imaging with small planar detectors. IEEE Nuclear Science Symposium Conference Record.

[49] Bal H, Guerin L, Casey ME, Conti M, Eriksson L, Michel C, Fanti S, Pettinato C, Adler S, Choyke P (2014). Improving PET spatial resolution and detectability for prostate cancer imaging. Phys Med Biol.

[50] Hausmann D, Bittencourt LK, Attenberger UI, Sertdemir M, Weidner A, Büsing KA, Wenz F, Schoenberg SO, Dinter DJ (2014). Diagnostic accuracy of ^18^F choline PET/CT using time-of-flight reconstruction algorithm in prostate cancer patients with biochemical recurrence.. Clin Nucl Med.

[51] Heck MM, Souvatzoglou M, Retz M, Nawroth R, Kübler H, Maurer T, Thalgott M, Gramer BM, Weirich G, Rondak IC, Rummeny EJ, Schwaiger M, Gschwend JE, Krause B, Eiber M (2014). Prospective comparison of computed tomography, diffusion-weighted magnetic resonance imaging and [^11^C] choline positron emission tomography/computed tomography for preoperative lymph node staging in prostate cancer patients. Eur J Nucl Med Mol Imaging.

[52] Haseebuddin M, Dehdashti F, Siegel BA, Liu J, Roth EB, Nepple KG, Siegel CL, Fischer KC, Kibel AS, Andriole GL, Miller TR (2013). ^11^C-acetate PET/CT before radical prostatectomy: nodal staging and treatment failure prediction. J Nucl Med.

[53] Takei T, Souvatzoglou M, Beer AJ, Drzezga A, Ziegler S, Rummeny EJ, Schwaiger M, Eiber M (2012). A case of multimodality multiparametric ^11^C-choline PET/MR for biopsy targeting in prior biopsy-negative primary prostate cancer.. Clin Nucl Med.

[54] Souvatzoglou M, Eiber M, Takei T, Fürst S, Maurer T, Gaertner F, Geinitz H, Drzezga A, Ziegler S, Nekolla SG, Rummeny EJ, Schwaiger M, Beer AJ (2013). Comparison of integrated whole-body [^11^C]choline PET/MR with PET/CT in patients with prostate cancer. Eur J Nucl Med Mol Imaging.

[55] Wetter A, Lipponer C, Nensa F, Heusch P, Rübben H, Altenbernd J-C, Schlosser T, Bockisch A, Pöppel T, Lauenstein T, Nagarajah J (2014). Evaluation of the PET component of simultaneous [^18^F]choline PET/MRI in prostate cancer: comparison with [^18^F]choline PET/CT. Eur J Nucl Med Mol Imaging.

[56] Reed GD, Larson PE, Morze C, Bok R, Lustig M, Kerr AB, Pauly JM, Kurhanewicz J, Vigneron DB (2012). A method for simultaneous echo planar imaging of hyperpolarized ^13^C pyruvate and ^13^C lactate.. J Magn Reson.

[57] Verma S, Turkbey B, Muradyan N, Rajesh A, Cornud F, Haider MA, Choyke PL, Harisinghani M (2012). Overview of dynamic contrast-enhanced MRI in prostate cancer diagnosis and management. AJR Am J Roentgenol.

[58] Turkbey B, Choyke PL (2012). Multiparametric MRI and prostate cancer diagnosis and risk stratification. Curr Opin Urol.

[59] Bouchelouche K, Turkbey B, Choyke P, Capala J (2010). Imaging prostate cancer: an update on positron emission tomography and magnetic resonance imaging. Curr Urol Rep.

[60] Hartenbach M, Hartenbach S, Bechtloff W, Danz B, Kraft K, Klemenz B, Sparwasser C, Hacker M (2014). Combined PET/MRI improves diagnostic accuracy in patients with prostate cancer: a prospective diagnostic trial. Clin Can Res.

[61] Sutinen E, Nurmi M, Roivainen A, Varpula M, Tolvanen T, Lehikoinen P, Minn H (2004). Kinetics of [^11^C]choline uptake in prostate cancer: a PET study. Eur Jour Nucl Med Mol Imag.

[62] Park H, Wood D, Hussain H, Meyer CR, Shah RB, Johnson TD, Chenevert T, Piert M (2012). Introducing parametric fusion PET/MRI of primary prostate cancer. Jour Nucl Med.

[63] Zakian KL, Shukla-Dave A, Ackerstaff A, Hricak H, Koutcher JA (2008). ^1^H magnetic resonance spectroscopy of prostate cancer: biomarkers for tumor characterization. Cancer Biomarkers.

[64] Piert M, Park H, Khan A, Siddiqui J, Hussain H, Chenevert T, Wood D, Johnson T, Shah RB, Meyer C (2009). Detection of aggressive primary prostate cancer with ^11^C-choline PET/CT using multimodality fusion techniques.. J Nucl Med.

