# Supplementary figures and images for: New prospects for PET in prostate cancer imaging: a physicist's viewpoint
Source: EJNMMI Phys. 2014 Sep 9;1:11. doi: 10.1186/2197-7364-1-11 (PMC6890878; doi:10.1186/2197-7364-1-11)

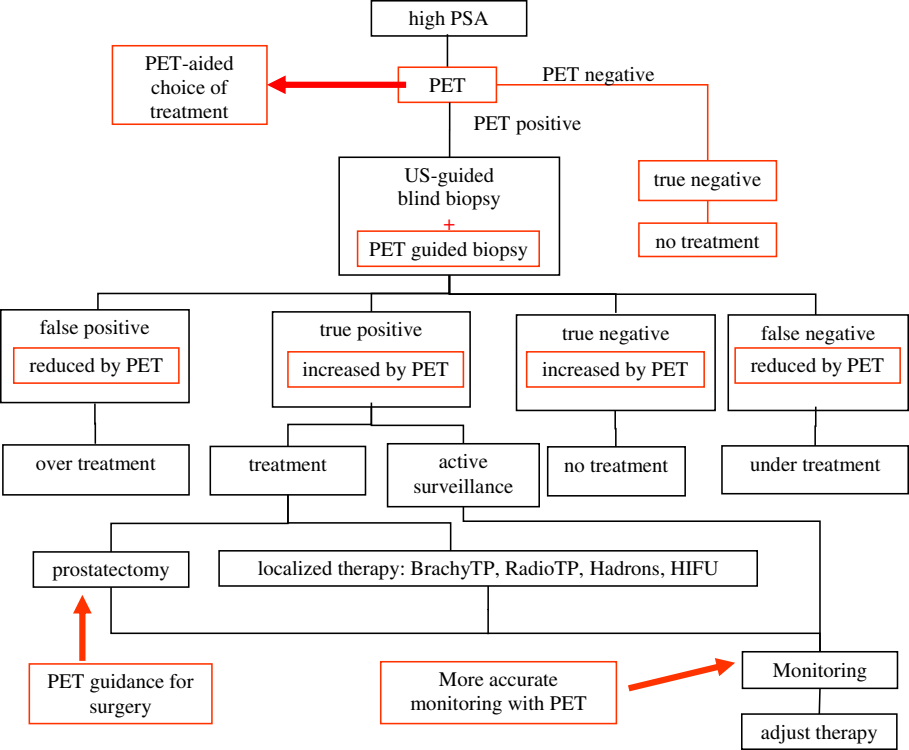

Supplement: Supplementary file 1 — Authors’ original file for figure 1 [file 40658_2014_9002_MOESM1_ESM.pdf]

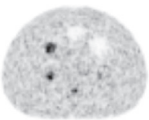

OSEM 4mm  
(a)

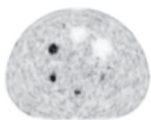

PSF 2mm  
(b)

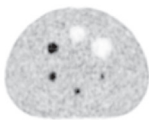

PSF+TOF 2mm  
(c)

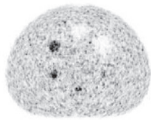

OSEM 2mm  
(d)

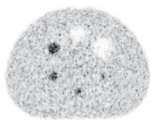

OSEM+TOF 2mm  
(e)

Supplement: Supplementary file 2 — Authors’ original file for figure 2 [file 40658_2014_9002_MOESM2_ESM.pdf]

a

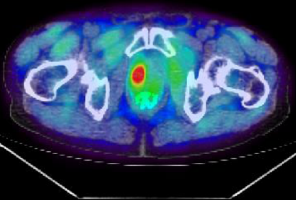

b

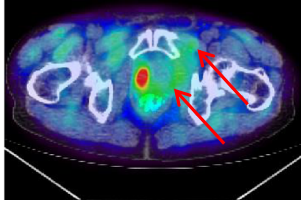

c

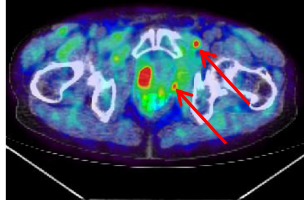

Supplement: Supplementary file 3 — Authors’ original file for figure 3 [file 40658_2014_9002_MOESM3_ESM.pdf]
